# Hypertension management in primary care: study protocol for a cluster randomized controlled trial

**DOI:** 10.1186/s13063-015-0627-z

**Published:** 2015-03-21

**Authors:** Birgitta Weltermann, Anja Viehmann, Christine Kersting

**Affiliations:** Institute for General Medicine, University Hospital Essen, University of Duisburg-Essen, Hufelandstr. 55, 45147 Essen, Germany

**Keywords:** cluster randomized trial, hypertension management, primary care, blood pressure control, quality improvement, practice redesign

## Abstract

**Background:**

Studies worldwide show insufficient blood pressure control rates, and effective management of hypertension remains a challenge in general practice. Although structured forms of care improved blood pressure in randomized controlled trials, little is known about their effects under routine primary care. This cluster randomized trial (CRT) evaluates the effects of a modern interactive medical education series for general practitioners on hypertension management, including practice redesign strategies.

**Methods/Design:**

For this CRT, 24 primary care academic teaching practices of the University of Duisburg-Essen, Germany, are randomized into two study arms. With the objective of improving hypertension control, general practitioners of the intervention group participate in a three-session medical education program on structured hypertension management. The program aims at changing physician awareness and practice design. Various practice tools are provided: for example, checklists on valid blood pressure readings, medication selection, detection of secondary hypertension, and patient education. General practitioners of both study groups include hypertensive patients with and without hypertension-related diseases such as angiographically proven coronary disease, and peripheral or cerebral vascular disease. Blood pressure is measured by 24-hour readings. Analyses will focus on differences in blood pressure control and changes of practice management between intervention and control group.

**Discussion:**

The study will determine the effectiveness of our practice redesign intervention on hypertension control. The intervention addresses general practitioners and practice assistants, while aiming at benefits on the patient level. Therefore, the cluster design is used to evaluate the effects.

**Trial registration:**

DRKS00006315 (date of registration: 14 July 2014).

## Background

According to a World Health Organization (WHO) report, hypertension is the single most important risk factor, accounting for 13% of mortality worldwide [1]. The EUROASPIRE III study documented that blood pressure control rates in patients with documented coronary artery disease averaged only 39% in eight European countries despite the availability of various therapeutic options [2]. A Cochrane review of 72 randomized controlled trials compared various interventions for controlling blood pressure: a complex intervention including patient recall systems and education aimed at overcoming physicians’ hesitancy to prescribe complex medication regimens was the best strategy, decreasing average systolic and diastolic blood pressure by 8.0 mmHg and 4.3 mmHg [3]. The randomized controlled Hypertension Detection and Follow-up Program (HDFP) study, which developed and applied this structured care for hypertensive patients, documented a significant decrease in mortality rates after 5 years: all-cause mortality in the intervention group was 17% lower than in the control group [4]. However, the challenge is still to translate these findings into routine care [3].

Internationally, primary care is delivered in various organizational settings ranging from one- or two-physician offices scattered in urban or rural areas to large-sized multi-physician practices and health maintenance organizations. Therefore, strategies to improve hypertension control are needed that are applicable in various clinical scenarios [3].

This cluster randomized trial evaluates the effectiveness of a modern medical education series for primary care physicians about structured hypertension management on blood pressure control in hypertensive patients. The intervention follows a modern didactic concept with evidence-based, interactive case discussions and practice redesign strategies. Primary care academic teaching practices are randomized into intervention group (education on hypertension management) and control group (no education).

## Methods/Design

### Study design

The study is designed as a cluster randomized trial with primary care practices as the unit of randomization. The cluster design is used because the intervention (education) addresses practice teams, and thereby aims to improve patient outcomes indirectly [5]. Additionally, this design takes into account that patients of a certain practice may be more homogenous regarding medical and sociodemographic characteristics than patients of other practices [5,6]. Each practice cluster includes five patients at minimum.

Practices from both arms will receive the intervention, but sequentially, with control practices receiving the intervention after the follow-up data collection in both study groups.

### Practice and patient recruitment

The study is conducted in the practice network of 180 primary care academic teaching practices of the University Duisburg-Essen, Germany. These practices are distributed in urban and rural regions of North Rhine-Westphalia with a distance of 5 to 150 kilometers to the Institute for General Medicine in Essen, Germany. All practices belong to the Association of Statutory Health Insurance Physicians North Rhine and Westphalia-Lippe. All practices are requested to take part in one of two network meetings of the institute yearly. Each practice is allowed to choose which meeting to attend: the spring or the fall meeting. All practices that participated in the spring meeting 2013 were asked to participate in this study (n = 51). Interested physicians are contacted by telephone to check practice inclusion criteria. Practices are eligible for participating in the study if they provide health services to hypertensive patients and are equipped with at least one calibrated 24-hour blood pressure monitoring device. A total of 24 practices volunteered for participation and fulfilled the inclusion criteria. These practices were randomized in two study arms of equal size. All participating physicians are asked to sign an informed consent form. Patients are recruited by these physicians.

### Inclusion and exclusion criteria for patients

Patients are eligible for recruitment if 1) they are ≥18 years old and 2) their blood pressure is uncontrolled according to current guidelines: ≥140/90 mmHg in office readings, and/or ≥130/80 mmHg in 24-hour ambulatory measurements (based on the average 24-hour blood pressure), and/or ≥135/85 mmHg in patient self-measurements at home (based on the mean of a one-week protocol with at least two measurements daily) [7]. Patients with and without hypertension-related diseases such as angiographically proven coronary disease, peripheral or cerebral vascular disease, and/or high risk conditions such as diabetes can be recruited. All patients must be able to give informed consent and to read and comprehend the German language. Patients fulfilling all of these criteria are asked for study participation, receive written information about the study, and sign an informed consent form. A total of 169 patients were enrolled at baseline; of these, 101 (59.8%) were associated with the intervention arm.

### Outcome measures

The primary endpoint of the study is the change of the blood pressure control rate, defined as percentage of patients with an average 24-hour blood pressure <130/80 mmHg. The key secondary endpoints are the changes in average systolic and/or diastolic blood pressure (in mmHg) and changes in practice-specific hypertension management stratified by the intervention status of the practice. All outcomes are measured at baseline (before the intervention) and at follow-up (3 months after the last education session) in both study arms. This results in an average follow-up period of 5 months per patient.

To determine the interventional effectiveness on the patients’ level, each study patient receives a 24-hour ambulatory blood pressure monitoring at baseline and at follow-up. To control for confounding, general practitioners complete a questionnaire on medical characteristics of each study patient (for example, diagnosis, comorbidities, medication, and differential diagnoses). Study patients complete a self-administered paper and pencil questionnaire on individual characteristics such as sociodemographic characteristics, risk factors (for example, physical activity and smoking behavior), medication adherence, and management of blood pressure self-readings at baseline and follow-up.

At baseline, general practitioners complete a questionnaire on personal characteristics (age, sex), medical degree(s), additional qualifications, and individual attitudes toward hypertension treatment (motivation and self-efficacy). To determine practice changes after intervention, the follow-up questionnaire focuses on changes in daily practice routines and determines which of the hypertension management strategies are implemented by the practices.

### Intervention group

General practitioners of the intervention group participate in a modern education on strategies of structured hypertension management in primary care. It is conducted as an in-class, three-session medical education program. Central elements of the education are 1) training on valid upper arm blood pressure readings; 2) evidence-based information on resistant hypertension, secondary hypertension, and modern pharmacotherapy, in the form of presentations and group discussions of actual cases; and 3) the introduction and distribution of practice tools to facilitate the long-term implementation of hypertension management. The following practice redesign tools are provided in print and electronic versions:

1. current hypertension guidelines [7],
2. 10-step checklist how to obtain standardized upper arm office blood pressure readings according to guidelines [7],
3. template of a blood pressure documentation sheet for serial documentation of blood pressure values by patients,
4. list of electronic blood pressure devices that carry the quality seal of the German Hypertension League,
5. prescription template detailing how to prescribe an electronic blood pressure device,
6. prescription template detailing how to prescribe relaxation training programs (progressive muscle relaxation and autogenous training),
7. checklists to assist in the diagnosis of secondary hypertension,
8. templates for specialist referral sheets for the work-up of secondary hypertension,
9. overview sheet specifying the mechanism of action of the various antihypertensives,
10. information sheets on blood pressure lowering effects (in mmHg) of different single and combined blood pressure lowering measures such as regular physical activity or intensified pharmacotherapy [3], and
11. patient information leaflet about hypertension.

All information sheets are written in an easily understandable language and designed in a clear layout so that they can be used for patient information and education. General practitioners and practice assistants participate in the session on blood pressure measurements, while all other sessions are offered to physicians only. The education sessions are supported by hypertension specialists from the German Hypertension League.

Participating general practitioners are free in their choice of diagnostic and therapeutic strategies. Patient responsibility remains solely in the hands of the general practitioner in charge. Due to the physician-centered approach, study patients receive an indirect intervention.

### Control group

General practitioners of the control group are offered the same education sessions, but only after completing the follow-up data collection in both study arms. Control group patients receive treatment as usual.

Despite this sequential design, it cannot be excluded that study participation, 24-hour blood pressure measurement at baseline and follow-up, and completing a questionnaire at baseline and follow-up might influence the physicians’ and patients’ awareness for hypertension in both study arms.

### Pilot testing

The concept of this structured hypertension management for primary care, which is introduced during the education sessions, was developed in one exemplary teaching practice in our practice network. It combines guideline-based therapy, patient information on physical activity and diet, checklists for work-up of secondary hypertension, and patient supervision on blood pressure self-measurements according to standard. Patients were instructed about the documentation of blood pressure readings and how to react in case of blood pressure extremes. Patients with uncontrolled hypertension were scheduled for repetitive appointments to adjust the medications until blood pressure was in the target range or until other factors limited control. A phone recall to reschedule an appointment was performed for patients who had missed an appointment for blood pressure control. The retrospective evaluation of this hypertension management concept used in the model practice demonstrated that this strategy is effective: within one year blood pressure control rate of high-risk hypertensive patients increased from 46% to 74% [8] and for hypertensive patients in primary prevention, blood pressure control rate increased from 35% to 60% [9]. The results of the pilot testing are published separately [8,9].

### Sample size calculation

For the primary endpoint, a blood pressure control rate of 35% is assumed for the control group, while a blood pressure control rate of 70% is assumed for the intervention group [8,9]. Assuming an equal number of clusters per study arm, an average cluster size of five patients and an intraclass correlation coefficient of 0.05 [5], ten clusters per study arm are needed to detect the assumed effect using a chi-square test with a two-sided significance level of 5% and a power of 80%. Sample size calculation was performed in R package CRTSize, function n4props [10].

### Quality control procedures

Quality controls are conducted during all study phases. After recruitment, physicians’ and patients’ inclusion criteria are controlled. Consent forms are checked for completeness. After baseline and follow-up data collection, quality controls are conducted to assure that required data collection documents are complete. Baseline and follow-up data of each study patient are checked using birth date to ensure that data are merged correctly.

The data are entered manually in an electronic database with restricted access. To control for input errors, 10% of the data are entered twice. An error rate of 5% is accepted; otherwise all values are entered twice. The data are checked for plausibility using simple frequencies.

### Statistical hypotheses, methods, and analyses

The primary endpoint (differences in the blood pressure control rate between intervention and control group) will be analyzed using a chi-square test. A *P* value <0.05 will be considered significant.

For analyses of secondary endpoints, average blood pressure levels at baseline and at follow-up will be determined for all study patients and separately for patients of both study arms. Differences between baseline and follow-up will be tested for significance within each study arm using paired t-tests. Differences in the mean systolic and diastolic blood pressure level of patients of the intervention group and patients of the control group will be tested for significance using an adjusted t-test for independent samples, which accounts for the intra-cluster correlation [6].

The analysis will determine which elements of hypertension management have been implemented into everyday routine during the study period and which of them are regarded as being helpful. To describe the effect of single hypertension management strategies on blood pressure, bivariate analyses will be conducted.

A generalized linear mixed model will be performed to identify predictors on blood pressure control at follow-up. The model will include patient level covariates such as age, sex, lifestyle, comorbidities, and changes in each patient’s hypertension management as well as cluster-level covariates such as changes of hypertension-related diagnostic and therapeutic strategies. Modeling will account for cluster effects by random effects modeling. Statistical analyses will be performed using IBM SPSS Statistics for Windows, Version 22.0 (Armonk, New York: IBM Corp.).

### Ethics and legal aspects

Study conduction complies with the ethical principles of the World Medical Association Declaration of Helsinki [11]. Ethical approval was obtained from the Ethic Commission of the Medical Faculty of the University of Duisburg-Essen (reference number: 13-5537-BO, date of approval: 9 September 2013). Participating general practitioners and patients sign informed consent forms. Physicians’ consent forms are sent to the coordinating institute. To guarantee patients’ anonymity, patients’ consent forms remain in the practices. The study materials referred to the study center at the Institute of General Medicine only contain the patients’ date of birth, yet other data allow patient identification.

## Discussion

Studies have identified various barriers for hypertension control in regular care that are structured in physician-, patient-, health care delivery and health care system-related factors [12]. Our cluster randomized trial is based on results from randomized controlled trials that show that complex interventions combining physician education, intensified pharmacotherapy and patient recall systems were most effective in improving hypertension control [3] and even decreased 5-year all-cause mortality [4]. Our intervention is using physician and practice assistant in-class education as the primary intervention, aiming at long-term hypertension control by modifying practice organization and patient education. Therefore, various hypertension management strategies for practice redesign are offered to physicians (so called ‘practice tools’) [13,14].

Cluster randomized trials from different countries evaluated various educational and organizational approaches to improve blood pressure control rates in primary care [15-24]. Most of these studies applied educational sessions for physicians and/or patients in combination with newly designed external support structures [15-22]. Three types of interventional approaches can be differentiated. The first category comprises patient-centered approaches, for example, used a clinical pharmacist led program [15,16] or electronic reminders on self-care to improve hypertension control [17]. A second category of studies evaluates physician-support structures, either by external study/audit centers or clinical pharmacists. These approaches span from feedback on pharmacotherapy by a pharmacist [18] to physician-pharmacist collaborations [19,20] to provider-specific audit reports with benchmarking elements [21] to physician-centered feedback with therapeutic recommendations by an external support structure to improve blood pressure therapy [22]. The effects vary from a mean group difference of +0.2 mmHg in systolic office blood pressure [22] to −10.3 mmHg in systolic 24-hour blood pressure [20] and from +0.04 mmHg in diastolic office blood pressure [21] to −4.6 mmHg in diastolic 24-hour blood pressure [19]. In comparison to these studies, only a few interventions aimed at internal practice redesign in addition to external support structures. Pouchain *et al*. introduced calibrated 24-hour blood pressure monitor devices in primary care practices, while Reuther *et al*. introduced strategies to organize hypertension management in primary care [23,24]. The mean group differences were −4.76/-1.88 mmHg [23] and −1.75/+0.14 mmHg [24]. In contrast to these studies, we are interested in redesigning primary care without external support structures addressing the physician manager in charge of the practice.

Interventions aimed at changing regular care need to be based on an in-depth understanding of the health care system that they are addressing [13]. We designed our intervention for the German primary care system, which is based mainly on one- or two- (rarely more) physician private practices that self-organize patient care and are regionally distributed in the various neighborhoods close to their patients. These practices typically serve sickness fund and privately insured patients likewise; therefore, they follow the regulations of the various insurance systems parallel. While all physicians and practice assistants in the intervention group will participate in the educational intervention to familiarize them with the array of options of a practice hypertension management, the practices remain free with regard to their choice of diagnostic, therapeutic and practice redesign strategies. Thereby, we follow a core principle of practice redesign that assumes that practice teams know best about their practice-specific contextual factors such as, for example, practice structures or organization [13]. Practitioners are more likely to pursue self-selected strategies than a fixed set of approaches.

Methodologically, we will address this expected inter-cluster variance by three strategies. First, for each practice we will obtain the hypertension management strategies at baseline and follow-up: this will allow us to assess which strategies are newly tried. In addition, the practices will be asked which strategies they will continue to use for their hypertension management. Second, based on this information we will outline typical patterns of hypertension management for inter-cluster analyses. Third, for each patient we will obtain the information in which changes in diagnostic, therapeutic and educational interventions occurred between baseline and follow-up. Depending on the frequencies and potential effects on hypertension control in bivariate analyses, we will include an appropriate selection of single measures or patterns of redesign aspects in the final regression model to describe the effects of the relevant hypertension management strategies on hypertension control in primary care patients.

### Trial status

The study began in September 2013. Baseline data collection was completed in January 2014. Follow-up data collection was completed in August 2014. Data management, including quality control, is ongoing. Results are expected in summer 2015.

## Abbreviations

CRT: cluster randomized trial
HDFP: hypertension detection and follow-up program
WHO: World Health Organization
